# The development of a patient-reported outcome measure for assessing nighttime symptoms of chronic obstructive pulmonary disease

**DOI:** 10.1186/1477-7525-11-104

**Published:** 2013-06-25

**Authors:** Asha Hareendran, Andrew C Palsgrove, Michelle Mocarski, Michael L Schaefer, Juliana Setyawan, Robyn Carson, Barry Make

**Affiliations:** 1 United BioSource Corporation, 26-28 Hammersmith Grove, Floor 5E, London W6 7HA, UK; 2 United BioSource Corporation, 7101 Wisconsin Avenue, Suite 600, Bethesda, MD 20814, USA; 3 Forest Research Institute, Harborside Financial Center, Plaza V, Jersey City, NJ 07311, USA; 4 National Jewish Health, 1400 Jackson St., Denver, CO 80206, USA

**Keywords:** COPD, Symptoms, PRO, Nighttime, Awakening

## Abstract

**Background:**

The assessment of symptoms of chronic obstructive pulmonary disease (COPD) is important for monitoring and managing the disease and for evaluating outcomes of interventions. COPD patients experience symptoms during the day and night, and symptoms experienced at night often disturb sleep. The aim of this paper is to describe methods used to develop a patient-reported outcome (PRO) instrument for evaluating nighttime symptoms of COPD, and to document evidence for the content validity of the instrument.

**Methods:**

Literature review and clinician interviews were conducted to inform discussion guides to explore patients’ nighttime COPD symptom experience. Data from focus groups with COPD patients was used to develop a conceptual framework and the content of a new PRO instrument. Patient understanding of the new instrument was assessed via cognitive interviews with COPD patients.

**Results:**

The literature review confirmed that there is no instrument with evidence of content validity currently available to assess nighttime symptoms of COPD. Additionally, the literature review and clinician interviews suggested the need to understand patients’ experience of specific symptoms in order to evaluate nighttime symptoms of COPD. Analyses of patient focus group data (N = 27) supported saturation of concepts and aided in development of a conceptual framework. Items were generated using patients’ terminology to collect data on concepts in the framework including the occurrence and severity of COPD symptoms, use of rescue medication at night, and nocturnal awakening. Response options were chosen to reflect concepts that were salient to patients. Subsequent cognitive interviewing with ten COPD patients demonstrated that the items, response options, recall period, and instructions were understandable, relevant, and interpreted as intended.

**Conclusions:**

A new PRO instrument, the Nighttime Symptoms of COPD Instrument (NiSCI), was developed with documented evidence of content validity. The NiSCI is ready for empirical testing, including item reduction and evaluation of psychometric properties.

## Background

Chronic obstructive pulmonary disease (COPD) is defined by persistent airflow limitation that is usually progressive and is associated with an enhanced chronic inflammatory response in the airways and the lungs to noxious particles or gases [1]. These changes result in breathlessness and other respiratory symptoms such as cough, sputum production, wheezing and chest tightness, which are associated with impaired quality of life of COPD patients [2]. In particular, sleep disturbances among patients with COPD have been linked to negative outcomes, underscoring the importance of both measuring and reducing sleep disturbance [3]. For instance, patients experience circadian variation in lung function and COPD symptoms occurring in the evening which may be particularly bothersome to patients due to their impact on sleep [3]. Similarly, a recent Internet survey showed that nighttime symptoms are common among patients with COPD, with 25% of patients indicating that their symptoms at night were worse than usual. Further, the experience of symptoms at night was even more pronounced among the subgroup of patients with severe COPD, with 34% reporting a worsening of symptoms at night [4].

Reducing symptom severity is a key target for the clinical management of COPD and recent guidelines encourage the use of validated questionnaires to assess COPD symptoms [1,5]. Only a few clinical trials for COPD maintenance therapies have specifically evaluated the benefit of treatment on nighttime symptoms of COPD, using methods such as daily diaries to record nighttime awakenings [6-10] and nighttime use of rescue medications [10,11]. However, there is currently no standardized approach to doing so.

In recent years, regulatory agencies have increased their scrutiny of patient-reported outcome (PRO) measures. In particular, the United Stated Food and Drug Administration (FDA) released the “Guidance for Industry on Patient-Reported Outcome Measures: Use in Medical Product Development to Support Labeling Claims,” which emphasizes the need to demonstrate an instrument’s content validity [12]. Achieving content validity requires concept elicitation from patients using qualitative methods; specifically, focus groups or individual patient interviews. Additionally, evidence of patient understanding of a measure is also important to ensure content validity [13,14]. This evidence is usually collected using cognitive interviewing following the completion of an initial draft of the instrument (initial draft referred to as Version 1 in this manuscript).

The aim of the present study was to develop a new PRO measure in accordance with the current FDA PRO Guidance [12], that could be used in clinical trials to support label claims pertaining to the benefit of treatments for nighttime symptoms of COPD (e.g., reduction of nighttime symptoms). This manuscript outlines the first phase of work done for concept elicitation to develop the content and format of the instrument and cognitive interviewing to test patients’ understanding of the new instrument, using methods outlined by the International Society of Pharmacoeconomic and Outcomes Research (ISPOR) PRO Good Research Practices Task Force [13,14].

## Methods

### Concept elicitation: literature review and clinician interviews

The research team conducted a review of the respiratory literature through EMBASE and MEDLINE databases (2000–2009), abstracts from the American College of Chest Physicians (2003–2008), the American Thoracic Society (2008–2009), and the European Respiratory Society (2005–2008). Information collected included articles and abstracts about: a) clinical trials which measured nighttime COPD symptoms, b) non-clinical trial literature that contained discussion of nighttime symptoms of COPD, and c) literature on other instruments measuring nighttime symptoms of COPD. Interviews with clinicians were also conducted in order to determine the clinical context of evaluating the benefit of treatment on nighttime symptoms of COPD. Information from this phase of the project was used to draft a framework for exploring patient experiences of nighttime COPD symptoms on which the subsequent instrument development work was based.

### Concept elicitation: focus group discussions

Focus group discussions were conducted to obtain information directly from COPD patients about the most important concepts related to the experience of COPD symptoms at night. A discussion guide was developed for the focus groups based on the conceptual framework resulting from the literature review and clinician interviews. The study protocol developed for concept elicitation (Version 3.0 dated 23rd December 2009) and cognitive interviews (Version 4.0 dated 4th September 2010) was reviewed and approved by a human subjects institutional review board (IRB).

Focus group participants were recruited from out-patient clinic sites in four different locations in the United States: Virginia, New York, North Carolina, and Texas. First, site staff were trained on the study protocol. Then, clinical research coordinators at the sites reviewed patient charts to determine potential patient eligibility. The research coordinators were provided with a screening script to introduce patients to the study, and were instructed to strive for representativeness among recruited patients that would be comparable to that of the general COPD patient population in terms of gender, age, ethnic diversity, and COPD severity. After patients reviewed the IRB-approved information about the study and provided written informed consent, research coordinators completed forms to document each patient’s eligibility and to capture information on clinical characteristics.

Focus group participants were selected to match the target population of future clinical trials assessing improvement in nighttime symptoms of COPD. Eligibility criteria for the focus groups were determined based on recommendations from clinical expert interviews. Thus, to be included in the study, focus group participants were required to have experienced COPD symptoms at night and/or the early morning for at least three nights/days a week, be 40 years of age or older, be a current or former cigarette smoker with a smoking history of at least ten pack-years, and have a current medical diagnosis of COPD (including chronic bronchitis and/or emphysema).

Participants were excluded from the study if they had experienced a COPD exacerbation or unexpected visit to the clinic, ER, or hospital and/or used prescription medication to treat an exacerbation within six weeks prior to eligibility assessment; used narcotics, sleep aids, sedating antihistamines, sedatives, monoamine oxidase inhibitors (MAOIs), or other medications known to affect daytime somnolence or sleep quality (as assessed by site clinical research coordinators); did not maintain regular day/night waking/sleeping cycles (e.g., night shift workers); chronically used oxygen therapy ≥15 hours/day; had a Body Mass Index (BMI) of 35 or above; or had a diagnosis of asthma, sleep apnea, or experienced nocturnal symptoms of gastroesophageal reflux disease (GERD) that were not controlled by treatment.

Focus group discussions were led by trained and experienced moderators who used a semi-structured interview guide designed to facilitate discussion and create consistency across sessions. A trained research assistant was also present during focus groups to take notes and assist with the session. Moderators elicited information from participants regarding their experiences of nighttime symptoms of COPD, using open-ended questions. After each focus group, the research team reviewed the notes taken and the adequacy of the interview guide and revised it to enhance data collection, if needed.

At the close of the focus group discussions, each participant completed health status and sociodemographic questionnaires, including the St. George’s Respiratory Questionnaire for COPD Patients (SGRQ-C) [15,16] and the Hospital Anxiety and Depression Scale (HADS) [17,18]. Each session (focus group discussion and questionnaire completion) lasted approximately 1.5 hours, and was audio-recorded and transcribed for analysis.

### Analysis of focus group discussions

A phenomenological approach was used to analyze transcripts of the audio-recordings, based on grounded theory, which refers to the inductive process of identifying analytical categories as they emerge from the data [19,20]. The analysis involved open, axial, and selective coding, and the phenomenological approach aimed to understand patient experiences of COPD symptoms at nighttime. This process is an emergent methodology that aids hypothesis generation and allows the researcher to uncover key concepts and insights from the data.

A coding dictionary was developed by examining transcript data for themes and subthemes. To begin, two researchers independently reviewed and coded one of the four transcripts. The two researchers met to discuss inter-rater agreement and refined the coding approach and dictionary for use in examining the remaining three transcripts. Each of the three remaining transcripts was coded independently by the two researchers, after which, the two researchers reviewed their findings. The researchers discussed disagreements and reconciled any divergent codes to determine the final coding assessment across all four transcripts. An iterative process was used to identify analytical categories, and to analyze the data to uncover key concepts and insights until no new codes or coding groups were required; i.e., until all text from the transcripts had been assigned to one or more codes. Focus group data were analyzed with the assistance of ATLAS.ti (version 5.0) software [21]. Data from questionnaires were summarized using descriptive statistics.

### Conceptual framework

Concepts emerging from the focus group discussions were tracked on tables following each discussion, to monitor saturation. Saturation was defined as the point where themes from prior discussions were echoed in later discussions and no new themes were introduced beyond those previously identified. An analysis of the data from the concept elicitation work with patients confirmed saturation of concepts. Subsequently, the initial framework of concepts identified from the literature and clinician interviews was refined into a conceptual framework for a new PRO instrument.

### Item generation

Items were developed for Version 1 of the measure to collect information about all concepts in the revised conceptual framework. The authors used an iterative process of drafting, evaluation, and revision to generate the items, response options, instructions, and recall period for the instrument. Item stems and response options were generated to reflect the terminology used by patients. Recall period was selected to best capture the full variability of the concepts.

The initial item pool was reviewed by an expert in cultural and linguistic translation. To create Version 1, grammatical edits and minor changes in wording were made to ensure readability and facilitate consistency in words and meaning across translations.

### Confirmation of content validity: cognitive interviews

Following development of the item pool, the items were formatted as Version 1 of the PRO instrument, administered to patients, and tested in one-on-one cognitive interviews to further confirm the content validity of the instrument. Cognitive interviews were conducted to assess the respondents’ understanding of the questionnaire and its items, in relation to the intended meaning and to evaluate comprehensiveness of the content to evaluate the target concept [14]. Recruitment was from a single clinical research office, and followed the same recruitment process used for focus groups. The study protocol was reviewed and approved by an IRB. Informed consent was obtained from each participant prior to discussion of study-related materials. Inclusion and exclusion criteria were the same as for the focus groups, with the exception that participants in the cognitive interviews could not have participated in the earlier focus group discussions.

Trained and experienced interviewers administered Version 1 of the Nighttime Symptoms of COPD Instrument (NiSCI) and conducted interviews using a semi-structured interview guide. The administration of Version 1 of the NiSCI for cognitive interviewing was designed to mimic the format that would be used in clinical trials.

Patients were included in two rounds of cognitive interviewing. Following interviews with the first set of participants, the results and the interview guide were reviewed. Minor alterations to the instrument were made based on patient feedback and the interview guide was refined for clarity. Revisions were based on participant comments, and consideration was also given to data gathered during concept elicitation focus groups. The remaining participants completed the revised instrument (Version 2) and were interviewed using the updated interview guide.

During cognitive interviews, patients’ comprehension of the instrument and the comprehensiveness of the items were assessed, and patients were asked to provide feedback on the instrument’s instructions, as well as the appropriateness of the recall period and response options. To evaluate patient understanding, interviews were conducted with attention to verbal and non-verbal cues from the participants. At the end of the interview, patients completed the SGRQ, HADS, and a sociodemographic questionnaire. Interviews were audio-recorded and transcribed for analysis.

### Analysis of cognitive interviews

Qualitative analysis software (ATLAS.ti version 5.0) [21] was used to analyze the transcripts of the audio-recordings, as it organized participant responses to each of the items. Similar to the process used to analyze the data from concept elicitation interviews, coders discussed and revised the coding schemes and definitions throughout the analysis process, until all text from the transcripts could be assigned to one or more codes.

Following analysis of the cognitive interviews, edits were made to Version 1 of the instrument. In order to document the development and refinement of each item in the NiSCI, an item-tracking matrix was developed including information about the development of the item, changes made following translatability assessment, changes following the first round of cognitive interviews, and the final version of items (Version 2) following the second round of interviews.

**Figure 1:**
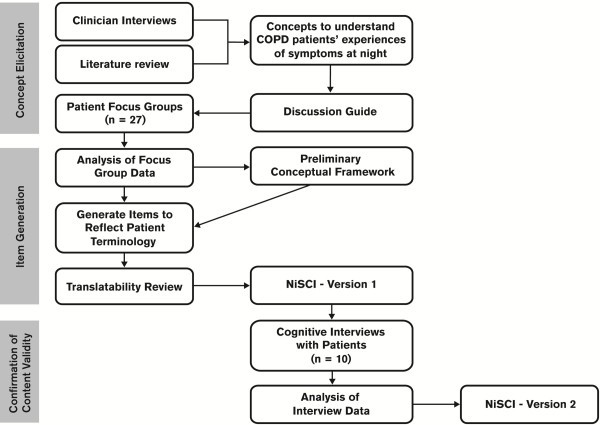
Overview of methods.

A schematic outline of the project methodology describing the steps for concept elicitation, item generation and cognitive testing can be found in Figure 1.

## Results

### Literature review and clinician interviews

Results of the literature review and clinician interviews (with five pulmonologists and a sleep expert) suggested that to understand patients’ experience of nighttime symptoms of COPD, it is important to evaluate their experience of COPD symptoms as well as the consequences of these symptoms on sleep. The results also informed the development of the protocol for these studies; for example, the necessary exclusion of conditions other than COPD that often result in the experience of respiratory symptoms at nighttime (e.g., sleep apnea). Additionally, the review of literature did not identify a PRO measure with documented evidence of content validity that could be used to collect data on nighttime symptoms of COPD. This suggested the need to develop a new tool.

### Description of the sample used for concept elicitation and cognitive interviewing

Table 1 shows the sociodemographic, health status, and clinical characteristics of the focus group and cognitive interview participants. While the goal was for the sample to reflect the characteristics of the study population to be included in clinical trials, a full range of COPD severity levels (GOLD I to IV) was included to ensure suitability of the content of the tool for all COPD patients.

**Table 1:** Sociodemographic characteristics of focus group and cognitive interview participants

**Characteristics**	**Focus groups N=27**	**Cognitive interviews N=10**
**Age, Mean (SD)**	68.1 (8.7)	68.1 (6.4)
**Gender, female, n (%)**	14 (51.9%)	7 (70%)
**Race/Ethnicity, n (%)**		
White	24 (88.9%)	5 (50%)
Black or African American	2 (7.4%)	5 (50%)
Native American or Alaska Native	1 (3.7%)	0 (0%)
Hispanic or Latino (not exclusive of race)	2 (7.4%)	0 (0%)
**Clinical information (as reported by clinicians at recruiting sites)**		
**GOLD stage, n (%)**		
I	2 (7.4%)	2 (20%)
II	15 (55.6%)	2 (20%)
III	4 (14.8%)	4 (40%)
IV	6 (22.2%)	2 (20%)
**Current smoker, yes, n (%)**	9 (33.3%)	2 (20%)
**SGRQ-C**^**a**^**, Mean (SD)**		
Total score	47.3 (18.6)	53.9 (16.3)
Symptom domain	57.7 (22.3)	62.9 (17.2)
Activity domain	65.9 (24.0)	76.5 (18.5)
Impact domain	33.1 (19.3)	37.5 (22.7)
**HADS**^**b**^**, Mean (SD)**		
Anxiety domain	4.7 (3.3)	7.0 (3.9)
Depression domain	4.4 (2.8)	5.2 (3.7)

GOLD, Global initiative for chronic Obstructive Lung Disease; HADS, Hospital Anxiety and Depression Scale; SD, standard deviation; SGRQ-C, St. George’s Respiratory Questionnaire for Chronic obstructive pulmonary disease.

^a^ SGRQ-C scores range from 0 to 100, with higher scores indicating poorer health status.

^b^ Domain scores range from 0 (no depression or anxiety) to 21; Scores of 8–10 suggest mild cases, 11–15 moderate cases, and 16 or above severe cases.

### Concept elicitation: focus group discussions

Twenty-seven patients with COPD participated in four focus groups. Of these, twenty-four reported experiencing COPD symptoms at night. During each focus group, participants discussed their experience in terms of the occurrence, severity, and impact of nighttime symptoms of COPD. Nighttime symptoms reported by the participants were shortness of breath (n = 17), cough (n = 15), mucus/phlegm (n = 14), and wheezing (n = 11). Additionally, three participants mentioned experiencing chest tightness, and two mentioned chest congestion. In the discussion, participants discussed the interrelationship among the different COPD symptoms. From their perspective, coughing, wheezing, congestion and mucus/phlegm production were related to each other. For example, patients described congestion being linked to both coughing and phlegm. No new COPD symptoms emerged in the fourth and final focus group, suggesting saturation of concepts (Table 2).

**Table 2:** Saturation grid: nighttime symptoms reported by patients during focus group discussions

**Symptom**	**FG1 n = 8**	**FG2 n = 7**	**FG3 n = 4**	**FG4 n = 8**
Wheezing	X		X	X
Coughing	X	X	X	X
Mucus or phlegm	X	X	X	X
Congestion			X	X
Shortness of breath	X	X	X	X
Tightness in chest			X	X
Headache	X			
Snoring	X			
Burning in chest	X			

FG, focus group.

Participants also discussed the impacts of nighttime symptoms (Table 3). In three of the four focus groups, participants described using inhaled rescue medication when their nighttime symptoms of COPD were worse than usual. Nighttime awakenings were also spontaneously mentioned in three of the four focus groups, with awakening being attributed to cough, wheezing, and shortness of breath. Some participants noted that they experienced COPD symptoms after awakening to use the bathroom, while others noted waking specifically due to COPD symptoms.

**Table 3:** Saturation grid: nighttime symptom severity

**Symptom**	**FG1 n = 8**	**FG2 n = 7**	**FG3 n = 4**	**FG4 n = 8**
Use of rescue inhalers	X		X	X
Nighttime awakening (unattributed)	X		X	X
Awakened by cough	X		X	X
Awakened by shortness of breath			X	X
Awakened by wheezing			X	

FG, focus group.

### Conceptual framework

The conceptual framework for the instrument was developed to depict the relationship of the various nighttime symptoms of COPD experienced by patients and the impact of these symptoms. Concepts that were clinically relevant and reported by patients, either through spontaneous report or following probing, were included in the conceptual framework. The clinical relevance of concepts was confirmed by referring to findings from the earlier clinician interviews and literature review, before finalizing the preliminary conceptual framework.

### Item generation

#### Content

The conceptual framework served as a basis for generating items for the new instrument. As a result, a 16-item instrument, the Nighttime Symptoms of COPD Instrument (NiSCI), was drafted in the form of a daily diary and included questions about the occurrence and severity of COPD symptoms, use of rescue medication at night, and nocturnal awakenings.

During the focus groups, participants discussed their experiences with specific symptoms at night. Thus, items were generated to collect information about each of these cardinal symptoms. Additionally, participants discussed their symptoms in terms of severity rather than frequency, therefore, response options were designed to cover the full range of symptom severity rather than assess symptom frequency. Items were not generated for other concepts reported by participants (headache, snoring, burning chest), as they were mentioned in only one of the four focus groups and by fewer than two participants for each concept. Additionally, none of these concepts were mentioned as being “COPD specific symptoms” in clinical guidance documents or during clinician interviews, and therefore, were not considered cardinal symptoms of COPD for evaluation of patients’ experiences of COPD symptoms at night.

As participants noted that they were woken up when symptoms worsened and sometimes had to use rescue medication, two items were generated: one to capture the occurrence of nocturnal awakening due to COPD symptoms and a second to assess the number of times rescue medication was used. Table 4 provides examples of patient quotes and the items that were generated based on them. A daily diary approach was selected to capture patient-perceived severity of symptoms and to help patients provide accurate and timely data about symptoms which vary from day-to-day in severity. To facilitate easier recall of nighttime experiences, the diary was designed to be completed at the beginning of their day. Patients were provided instructions to report on their experiences during the time period when they “went to bed to the time they woke up and got out of bed to start their day.”

**Table 4:** Example items following elicited concepts

**Concept**	**Quotes**	**Items**
Coughing	“I cannot lay down flat without coughing my head off.”	You indicated that you experienced a cough last night…
	“Well, I have mucous coughs, too, you know where you got to cough up something, but most of the time it’s not the mucous. It’s just a burning sensation right in my chest.”	- How severe was your cough?
Shortness of breath	“You’re calling for a breath to fill your lungs, but you can’t seem to get it.”	You indicated that you experienced shortness of breath last night…
	“It feels like I can’t get enough breath, and then when I get the breath, I-I know I have a hard time trying to get rid of it.”	- How severe was your shortness of breath?
Severity of symptoms	“I’m saying that I just get them or some of them I already have that would actually get worse. Uh, maybe sometimes I’ll have a relatively, uh, mild case of wheezing, but if I get real infectious, uh, I’m wheezing a lot. Uh, then I’ll be coughing a lot, so it-it’s hard to break down as to when, you know. It’s just hard to explain.”	Response options categorized as:
		• Mild
		• Moderate
		• Severe
		• Very Severe

To ensure data quality, the diary was designed to be incorporated on an electronic device (e.g., smart phone or personal digital assistant [PDA] for data collection in clinical trials).

#### Cognitive Interviews

To mimic the layout of the electronic device that will be used in clinical trials, each question or set of questions intended to appear on a single screen was presented to the patient on a single sheet of paper. Planned skip patterns for the instrument were also simulated by the interviewer, with follow-up items being presented based on the participants’ previous response. To ensure patients’ feedback on all of the questions in the instrument, if an item was skipped while the participant was completing the diary as a result of the skip patterns, it was later shown to the participant during the think-aloud portion of the interview. This process ensured that all patients were able to review and comment on all items, regardless of how they completed the instrument. Four patients were interviewed for the first stage of testing, and revisions made based on feedback from patients during this stage were tested with six additional patients.

Participants demonstrated an understanding of all instructions and items as intended, and expressed that no aspect of their experience of COPD symptoms at night were missing. Participants reported that they were able to recall their experiences of the previous night as they responded to each item, selecting the response that best represented their experiences. Cognitive interviews supported evidence of patient understanding of the response options.

In the first stage of testing, feedback from two participants suggested that further clarification regarding the recall period should be added to the instructions and definitions be explained to patients during patient training. However, timeframe to which the instructions referred (the previous night) was generally understood. Based on this feedback, additional clarifications were added to the instructions and it was noted that the timeframe should be stressed during site and patient training.

There was one minor revision made to an item’s response choices following the second stage of interviews to create Version 2 of the instrument: question marks after the symptoms listed in an item were removed for improved clarity on the screen of an electronic device.

## Discussion

This manuscript outlines the development of the NiSCI, an instrument to measure nighttime symptoms of COPD, and documents evidence of its content validity. This instrument is intended for future use in clinical trials to collect data to support label claims related to improvements in nighttime symptoms of COPD.

Consistent with the methods [13,14] required to ensure the content validity of PRO tools used to collect data to support claims [12], the development of the NiSCI was based on concept elicitation with patients. Patient understanding of the instrument was tested using cognitive interviewing. The conceptual framework for the instrument was based on concept elicitation from patients about their experiences of nighttime symptoms of COPD, with clinical validity ensured by referring back to initial information learned from the literature and clinician interviews.

Item development was guided by the conceptual framework and particular attention was paid to the incorporation of the terminology used by patients. Finally, cognitive interviews with patients following completion of the measure provided evidence that participants interpreted the items, instructions, and response options as intended, and that the items comprehensively evaluated patient experiences of nighttime symptoms of COPD.

This qualitative research showed that patients with COPD experience specific symptoms at night, and that the severity of these symptoms is variable across nights. The symptoms that patients described at night were consistent with the cardinal symptoms outlined in the COPD treatment guidelines (e.g., cough, sputum production [mucus and phlegm], shortness of breath) [22] and other symptoms known to be associated with COPD such as wheezing and chest tightness [22]. Additionally, the symptoms described by patients during the qualitative work were consistent with those reported by the clinicians interviewed for this study.

Patients noted throughout the discussions that their COPD symptoms often caused them to wake up during the night. They were able to report nocturnal awakening resulting from the experience of COPD symptoms as distinct from awakening for other reasons (e.g., to go to the bathroom). Research studies in COPD have linked sleep disturbances to negative outcomes, underscoring the importance of both measuring and reducing sleep disturbance [4]. However, in spite of the importance of sleep disturbances on patients’ well-being, to date nighttime awakenings have been infrequently studied as an endpoint in trials evaluating COPD treatments [6-10].

Focus group participants also discussed the need to use rescue medication when their nighttime symptoms increased in severity. This is consistent with the clinician interviews conducted for this study as well as COPD trials to date, as rescue medication use has often been used as an endpoint in clinical trials of products evaluated for the management of respiratory diseases [10,11].

The methods used for concept elicitation and cognitive interviewing closely followed the requirements for generating evidence of content validity for PRO tools, to be used to collect data to support label claims [12].

Though the sample sizes for the focus groups and interviews were small, the results showed evidence of saturation of concepts. A limitation of the concept elicitation work was that despite attempts to recruit a diverse group of patients, the final sample was not as diverse as intended, particularly in terms of race and ethnicity. More racial diversity was present in the sample of participants in the cognitive interviews. Subsequent testing of the instrument will also seek to include a diverse sample that represents the COPD population in the United States.

## Conclusions

Previous studies have demonstrated the importance of nighttime symptoms of COPD on various aspects of patients’ lives [2-4]; however there is currently no standardized way to measure these symptoms in clinical studies to examine potential treatment benefit. This study documents evidence of content validity for the NiSCI, a new PRO instrument to evaluate COPD nighttime symptoms. Future research will involve item reduction, evaluation of the instrument’s psychometric properties, and exploration of additional impacts of nighttime symptoms of COPD. Our goal is to convert the instrument to an electronic format and determine usability of the electronic version for clinical trials. Consistent with the methodological requirements to ensure content validity, the current conceptual framework will continue to be updated once item reduction and validation analyses are complete.

## Abbreviations

BMI: Body mass index; COPD: Chronic obstructive pulmonary disease; FDA: Food and Drug Administration; GERD: Gastroesophageal reflux disease; HADS: Hospital Anxiety and Depression Scale; IRB: Institutional review board; ISPOR: International Society of Pharmacoeconomic and Outcomes Research; MAOI: Monoamine oxidase inhibitor; NiSCI: Nighttime Symptoms of COPD Instrument; PDA: Personal digital assistant; PRO: Patient-reported outcome; SD: Standard deviation; SGRQ-C: St. George’s Respiratory Questionnaire for COPD.

## Competing interests

This study was conducted by United BioSource Corporation (UBC) on behalf of Forest Research Institute, Inc. (FRI), a wholly owned subsidiary of Forest Laboratories, Inc., who funded this work. Asha Hareendran, Andrew C. Palsgrove and Michael L. Schaefer as employees of UBC served as paid consultants to FRI during the conduct of this study and the development of this manuscript. Asha Hareendra is currently employed by UBC. Andrew C. Palsgrove is currently employed by Covance Market Access Services while Michael L. Schaefer is currently employed by Humana, Inc. Covance Market Access Services and Humana, Inc. were not associated with this study. Michelle Mocarski and Robyn Carson are employees of FRI. Juliana Setyawan worked for FRI while performing this analysis but is currently employed by Shire Pharmaceuticals. Shire Pharmaceuticals was not associated with this study. Barry Make received funds from UBC for consultation during development of the instrument.

## Authors’ contributions

AH led the design of the study and oversaw all aspects of the study including conduct of focus groups and patient interviews, data analysis, development of the draft instrument, and drafting of the manuscript. AP participated in the design of the study, conducted focus groups and patient interviews, analyzed the data, helped in the development of the draft instrument, and led the drafting of the manuscript. MM participated in the design of the study, interpretation of the data, development of the draft instrument, and drafting of the manuscript. MS participated in the design of the study, interpretation of the data, development of the draft instrument, and drafting of the manuscript. JS conceived of the study, participated in its design and coordination, development of the draft instrument, and drafting of the manuscript. RC participated in the design of the study, interpretation of the data, development of the draft instrument, and drafting of the manuscript. BM provided clinical expertise to the team and participated in the design of the study, provided feedback during instrument development, and participated in drafting of the manuscript. All authors read and approved the final manuscript.

## Authors’ information

Andrew C. Palsgrove worked for United BioSource Corporation during the implementation and reporting of this study but is currently employed by Covance Market Access Services. Covance Market Access Services was not associated with this study.

Michael L. Schaefer worked for United BioSource Corporation during the implementation and reporting of this study but is currently employed by Humana, Inc. Humana, Inc. was not associated with this study.

Juliana Setyawan worked for Forest Research Institute while performing this analysis but is currently employed by Shire Development LLC. Shire Development LLC was not associated with this study.
